# Superior Mesenteric Vein Thrombosis as a Rare Complication of Appendicitis: A Report of Two Cases

**DOI:** 10.7759/cureus.35794

**Published:** 2023-03-05

**Authors:** Zeyad Tareq Jaleel, Vinu Mathew

**Affiliations:** 1 Radiology, Hamad Medical Corporation, Doha, QAT

**Keywords:** emergency, computed tomography, superior mesenteric vein, thrombosis, acute appendicitis

## Abstract

Intra-abdominal inflammatory conditions, including acute appendicitis, are a common occurrence in the emergency department. In addition to employing various imaging modalities to determine the underlying cause, the consequences of these inflammatory diseases must be assessed. Thrombosis of the superior mesenteric vein is a rare complication of acute appendicitis. It is essential to be aware of this complication as early diagnosis may improve patient prognosis given that this consequence has a high mortality rate.

## Introduction

The examination of the computed tomography (CT) scan of the abdomen and pelvis to confirm the diagnosis of abdominal inflammatory illnesses such as acute appendicitis is one of the frequent clinical situations encountered in the emergency radiology department. One of the rare complications of an abdominal inflammatory infection is superior mesenteric vein thrombosis [1]. It requires prompt diagnosis as it carries high morbidity and mortality [2], which can be due to complications such as bowel ischemia and bowel infarction caused by mesenteric vein occlusion [3]. CT is the imaging modality most frequently used to diagnose the cause of intra-abdominal infection and its rare complications. In this article, we present two cases of superior mesenteric vein thrombosis, which is a rare complication of acute appendicitis that requires immediate identification and management.

## Case presentation

Case 1

A 34-year-old man who had been healthy before came to the hospital. He was febrile with a temperature of 38.9 degrees Celsius and had right-sided abdominal pain, vomiting, and a loss of appetite for two days. He denies having diarrhea or seeing blood in his stools. On physical examination, rebound tenderness was discovered in the right lower quadrant. Initial vital signs showed a heart rate of 85 BPM, a respiratory rate of 19 breaths per minute, a blood pressure reading of 125/82 mmHg, and an oxygen saturation of 99%. Initial laboratory testing revealed a high C-reactive protein at 264.3 mg/L and an elevated white blood cell count at 14.8 x 10^3^ cells/mcL.

**Figure 1:**
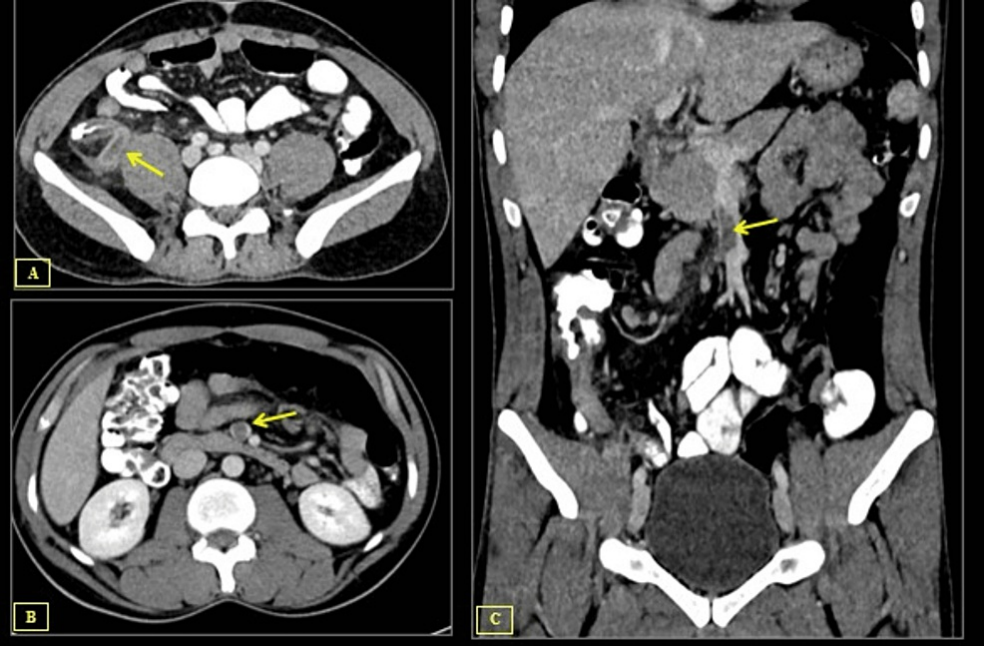
Multiple sections of axial and coronal contrast-enhanced CT of the abdomen in the portal venous phase (A) Axial contrast-enhanced CT abdomen shows an enlarged and thick-walled appendix (arrow), along with evidence of surrounding fat stranding. (B) Axial contrast-enhanced CT abdomen reveals a low attenuation thrombus within the superior mesenteric vein lumen (arrows). (C) Coronal contrast-enhanced CT abdomen reveals a low attenuation thrombus within the superior mesenteric vein lumen (arrows).

A CT of the abdomen and pelvis was done with oral and IV contrast, and it revealed a dilated, thickened, enhanced-walled appendix measuring 10.6 mm with abundant periappendicial fat stranding and surrounding free fluid (Figure 1A). Additionally, a filling defect that is indicative of thrombosis was identified in the superior mesenteric vein (Figures 1B, 1C).

Acute appendicitis was verified by histology after the patient was transported to the operating room for the procedure. Antibiotics and IV heparin with bridging to oral warfarin to attain therapeutic dosage were also started for management. The patient, however, did not come for his scheduled follow-up session.

Case 2

A previously healthy 41-year-old man arrived at the hospital with a 10-day history of nausea, vomiting, and abdominal pain. On physical examination, he was febrile, with a temperature of 38.3 degrees Celsius. He had tenderness in the epigastric and right paraumbilical regions, along with rebound tenderness in the right iliac fossa. Initial vital signs revealed an elevated heart rate of 115 BPM, a respiratory rate of 18 breaths per minute, a blood pressure reading of 120/82 mmHg, and an oxygen saturation of 99%. C-reactive protein levels were high at 272.4 mg/L, and white blood cell counts were slightly elevated at 11.2 x 10^3^/mcL.

**Figure 2:**
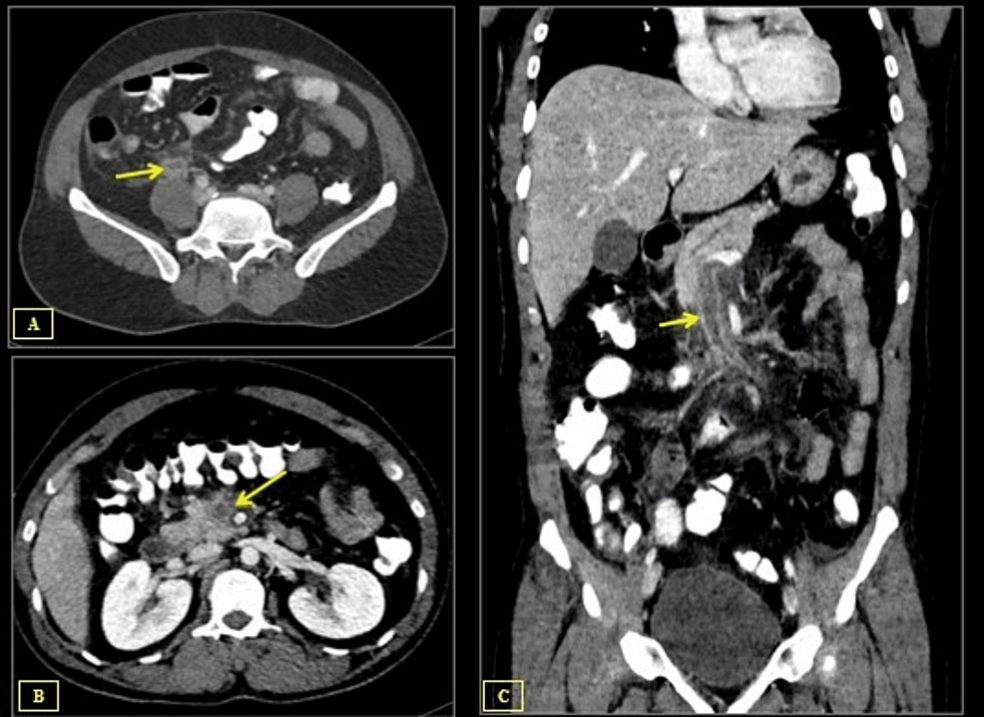
Multiple sections of axial and coronal contrast-enhanced CT of the abdomen in the portal venous phase (A) Axial contrast-enhanced CT abdomen shows an enlarged and thick-walled appendix (arrow), along with evidence of surrounding fat stranding. (B) Axial contrast-enhanced CT abdomen reveals a low attenuation thrombus within the superior mesenteric vein lumen (arrows). (C) Coronal contrast-enhanced CT abdomen reveals a low attenuation thrombus within the superior mesenteric vein lumen (arrows).

An enlarged appendix (Figure 2A) with the development of an appendicular mass was visible on a CT scan of the abdomen and pelvis with oral and IV contrast. Additionally, there was a filling defect that was seen in the superior mesenteric vein and its distal tributaries, reaching proximally to the portal vein confluence and suggesting thrombosis (Figures 2B, 2C).

The surgeon chose to use anticoagulation and antibiotics as a conservative approach. The patient showed clinical improvement and nearly full laboratory test result normalization after a two-week treatment of antibiotics and anticoagulation. At subsequent outpatient visits, the patient’s clinical condition had improved, and because he had achieved the initial target of three months of anticoagulation, the medication was withdrawn.

## Discussion

One of the rare complications of intraabdominal inflammatory infections like appendicitis that cause high morbidity and mortality is superior mesenteric vein thrombosis [1]. The other most commonly reported etiology is pancreatitis, followed by diverticulitis, peritonitis, and, less commonly, cholecystitis [4]. Adjacent to the infection, there is the propagation of the thrombus from the smaller veins that drain into the superior mesenteric vein and portal vein [5]. This thrombosis can cause occlusion of the SMV, which may lead to bowel ischemia and infarction, which is one of the causes of its high morbidity and mortality [6]. Imaging modalities like CT scans can help by not only detecting the primary source of infection but also assessing the rate of complications such as thrombosis and its extent [7,8].

There have been no clear, concise, randomized controlled studies to help guide the treatment of superior mesenteric vein thrombosis, but the consensus is to remove the source of infection if surgically possible, followed by medical management with antibiotics and anticoagulation therapy. Though anticoagulation was not recommended in the previous articles, there have been several studies that demonstrate lower mortality with anticoagulant patients [4,5,9,10], as well as studies that show early use of anticoagulants increases the rate of thrombus resolution or recanalization [4,9,11]. Another study that suggested the use of systemic anticoagulants was by Naymagon et al. [12], who found that anticoagulation significantly increased the rate of thrombus resolution and lowered the incidence of chronic symptomatic portal hypertension in their study of 67 patients.

## Conclusions

When managing intraabdominal acute disorders such as appendicitis, we should also care for complications such as superior mesenteric vein thrombosis, as demonstrated in this instance. These are obviously evident on imaging, and prompt diagnosis and treatment are necessary to improve the patient's prognosis. The recommended treatment is to find and control the source of the infection, then give antibiotics and anticoagulant medication as soon as possible.
